# Relation Between Working Memory Capacity of Biological Movements and Fluid Intelligence

**DOI:** 10.3389/fpsyg.2019.02313

**Published:** 2019-10-18

**Authors:** Tian Ye, Peng Li, Qiong Zhang, Quan Gu, Xiqian Lu, Zaifeng Gao, Mowei Shen

**Affiliations:** ^1^Department of Psychology, Zhejiang University, Hangzhou, China; ^2^School of Education and Management, Yunnan Normal University, Kunming, China

**Keywords:** biological motion, working memory, fluid intelligence, PLDs, IQ

## Abstract

Studies have revealed that there is an independent buffer for holding biological movements (BM) in working memory (WM), and this BM-WM has a unique link to our social ability. However, it remains unknown as to whether the BM-WM also correlates to our cognitive abilities, such as fluid intelligence (Gf). Since BM processing has been considered as a hallmark of social cognition, which distinguishes from canonical cognitive abilities in many ways, it has been hypothesized that only canonical object-WM (e.g., memorizing color patches), but not BM-WM, emerges to have an intimate relation with Gf. We tested this prediction by measuring the relationship between WM capacity of BM and Gf. With two Gf measurements, we consistently found moderate correlations between BM-WM capacity, the score of both Raven’s advanced progressive matrix (RAPM), and the Cattell culture fair intelligence test (CCFIT). This result revealed, for the first time, a close relation between WM and Gf with a social stimulus, and challenged the double-dissociation hypothesis for distinct functions of different WM buffers.

## Introduction

Biological movements (BMs) refer to the movements of animate entities (Johansson, 1973). Researchers have demonstrated converging evidence that BM contains abundant social information; for example, identity, gender, social interaction, intention, and emotion can be extracted from BM (e.g., Pollick et al., 2002; Atkinson et al., 2004; Petrini et al., 2014; for reviews see Puce and Perrett, 2003; Blake and Shiffrar, 2007; Troje, 2013). The ability to successfully and efficiently process human BM is critical to being a functioning member of human society (e.g., Perry et al., 2010; Herrington et al., 2011; Pavlova, 2012; Troje, 2013; Cook et al., 2014; Ding et al., 2017; Thornton, 2018), and healthy adults are considered experts at processing BM (Johansson, 1973; Fox and McDaniel, 1982; Troje, 2013).

Our cognitive system even involves an independent buffer for processing BMs (Smyth et al., 1988; Smyth and Pendleton, 1989, 1990; Wood, 2007, 2008, 2011; Cortese and Rossi-Arnaud, 2010; Shen et al., 2014; Liu et al., 2019) in working memory (WM), which maintains and manipulates a limited amount of information for the ongoing tasks (Baddeley and Hitch, 1974; Cowan, 2010). Just as there is a WM module specific for object or spatial information (i.e., object-WM vs. spatial-WM; Baddeley, 1996), there is also a WM module dedicated to BM information (Smyth et al., 1988; Smyth and Pendleton, 1989; Wood, 2007, 2008, 2011; Shen et al., 2014; Liu et al., 2019). Previous studies explored the BM-WM mechanisms by using real human movements (Smyth et al., 1988; Smyth and Pendleton, 1990; Wu and Coulson, 2014), computer-generated animations of human movements (Wood, 2007, 2011), imaginary BMs by the given names (Cortese and Rossi-Arnaud, 2010), and point light displays (PLDs) of human movements (Shen et al., 2014; Liu et al., 2019). For instance, Wood (2007) and Shen et al. (2014) demonstrated that participants could simultaneously hold a set of BM and a set of visual objects (e.g., colors) or locations in WM without significant mutual impairments. Liu et al. (2019) further found that memorizing BM was not modulated by the number of concurrent retained feature bindings in WM, and vice versa. Meanwhile, as compared to object-WM, BM-WM only holds a maximum of 3–4 BM stimuli (Smyth et al., 1988; Wood, 2007; Shen et al., 2014).^1^ Later neuroimaging studies further uncovered the neural substrates of BM-WM by showing that the mirror neuron system (MNS) plays a pivotal role in retaining BM in WM (Gao et al., 2013; Lu et al., 2016; Cai et al., 2018). Recent studies have also begun to explore issues such as the development of BM-WM (He et al., 2019), the influence of other social information (e.g., social interaction and emotion) on BM-WM capacity (Ding et al., 2017; Guo et al., 2019), BM-related binding in WM (Wood, 2008; Poom, 2012; Ding et al., 2015; Gu et al., 2019), the representation format of BM in WM (Vicary and Stevens, 2014; Vicary et al., 2014), and the frame of reference for remembering BM (Wood, 2010).

Although working memory^2^ capacity is rather limited, ample studies have consistently revealed that WM capacity has substantial predictive power in terms of predicting performance of high-level cognitive activities, including reading abilities, scholastic aptitude, information selection, and fluid intelligence (Gf) (e.g., Conway et al., 2002; Woodman et al., 2007; Hollingworth et al., 2008; Unsworth et al., 2014). Among these intimate relations, the relation between WM and Gf has received particular attention. Gf refers to the abilities needed for abstract reasoning and speeded performance (Cattell, 1971). In the last 15 years, researchers have revealed that the WM capacity of visual objects (e.g., color, shape; the corresponding WM buffer is named as object-WM) can significantly predict an individual’s Gf (Kane et al., 2005; Fukuda et al., 2010; Unsworth et al., 2014; Hicks et al., 2015). However, no study thus far has investigated the relation between BM-WM capacity and Gf, the answer to which will significantly improve our understanding about the processing mechanism and the function of BM-WM.

On the one hand, there might be no relation between WM capacity of BM and Gf. It has been claimed that the recognition ability to process BM is a hallmark of social cognition (Pavlova, 2012). Neuroimaging studies have revealed that the human MNS, which serves as key neural substrates for social activities such as mentalizing and empathy (e.g., Gallese and Goldman, 1998; Hooker et al., 2008; Shamay-Tsoory et al., 2009; Liakakis et al., 2011; Grecucci et al., 2013; Sperduti et al., 2014), plays a pivotal role not only in visual perception of BM (Saygin et al., 2004; Pavlova, 2012; Gilaie-Dotan et al., 2013), but also in retaining BM in WM (Gao et al., 2013; Lu et al., 2016). Moreover, recent WM studies implied that even merely retaining a frame of BM (e.g., static hand gestures) in WM, the MNS is also involved (Galvez-Pol et al., 2018a, b; Arslanova et al., 2019). Since the BM-WM buffer is suggested to play an important role in transferring ongoing social information from perception to WM (Urgolites and Wood, 2013; Shen et al., 2014), it is possible that the BM-WM capacity could inherently predict one’s social ability instead of the general cognitive ability (e.g., Gf). Corroborating this possibility, we recently found that BM-WM capacity positively correlated with both empathy (Gao et al., 2016) and theory of mind score (He et al., 2019), whereas such a relation vanished for WM capacity of movements of rectangles (i.e., non-animate motion) or of colors (i.e., object-WM). Because of the intimate relation between BM and social ability in both perception and WM, it has been suggested that BM-WM is a representative of social WM (He et al., 2019), which maintains and manipulates a limited set of social information in an online manner and is of paramount importance for navigating our social environment (Meyer and Lieberman, 2012), and is the best manner to measure the development of social WM in preschoolers (He et al., 2019). Critically, previous studies have only addressed whether there was a link between BM-WM and social ability, but no study has examined whether BM-WM is constrained to social ability. In other words, whether BM-WM capacity has no predictive power over Gf needs to be elucidated. If a null result is revealed, we then find a double dissociation in terms of different roles of WM buffers, with object-WM closely linking to canonical cognitive ability and BM-WM correlating to social ability.

On the other hand, since the storage of WM involves a series of cognitive operations, WM may have a tight relation with Gf, regardless of the stimuli type. Two recent functional magnetic resonance (fMRI) studies (Lu et al., 2016; Cai et al., 2018) found that, in addition to the MNS, the superior and inferior parietal lobule (SPL and IPL) and bilateral prefrontal cortex, which contribute to general cognitive processes (e.g., Todd and Marois, 2004; Xu and Chun, 2006; Barbey et al., 2013), also play a role in retaining BM in WM. Therefore, it is also possible that BM-WM capacity not only correlates to social ability, but also links to general cognitive ability.

The current study thus attempted to elucidate whether BM-WM has a close relationship with Gf. BM-WM was measured by using PLDs stimuli (Johansson, 1973). To ensure the validity of our study and facilitate comparisons with previous research, we adopted the widely used Raven advanced progressive matrices (RAPM) and Cattell cultural fair intelligence test (CCFIT) as our Gf measurements (Kane et al., 2005; Fukuda et al., 2010; Unsworth et al., 2014).

## Pilot Study

We first conducted a pilot study with 60 participants to estimate the potential correlation coefficients between BM-WM and the Gf measurements. Results from the pilot study were then used for calculating the final sample size with 90% power on a 0.05 significant level.

### Method

#### Participants

A total number of 60 participants took part in the pilot study. Thirty (18 males; mean ± SD age 21.3 ± 2.04 years) participants were from Zhejiang University, and thirty (15 males; mean ± SD age 18.9 ± 1.03 years) were from Yunnan University and Yunnan Normal University. Participants all had normal color vision and normal or corrected-to-normal visual acuity and received payment/course credit for their participation. Before the experiment, participants provided signed informed consent. The study was approved by the Research Ethics Board of Zhejiang University, Yunnan University and Yunnan Normal University.

#### Stimuli and Apparatus

**FIGURE 1:**
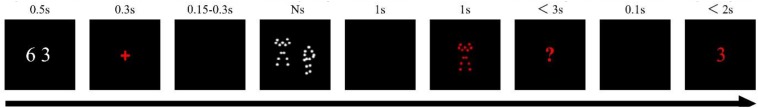
A schematic demonstration of procedures for measuring BM-WM capacity.

For the BM-WM test, PLDs were used as the BM stimuli. For each PLDs movement, 13 light points are placed at distinct joints of a moving human body to form a coherent and meaningful movement. We adopted PLDs in order to isolate BM information from other sources (e.g., color, contour, and texture; for a review see Troje, 2013). Seven movements were selected from the Vanrie and Verfaillie (2004) database: cycling, jumping, painting, spading, walking, waving, and chopping (see Figure 1). 30 distinct frames consisted one animation with each animation displayed twice consecutively, leading to a 1-s animation (refresh rate, 60 Hz). Each stimulus subtended a visual angle of approximately 1.64° × 1.64° from a viewing distance of 60 cm. In line with previous studies measuring BM capacity (e.g., Shen et al., 2014; Gao et al., 2016), during each trial one to five distinct stimuli would show up randomly on the periphery of an invisible circle (radius, 4.88° from the screen center) evenly.

For Gf measurement, two solidly validated Gf questionnaires were adopted: the Cattell culture fair intelligence test (CCFIT) and Raven’s advanced progressive matrix (RAPM). These two questionnaires were chosen for two considerations. First, both are non-verbal tests, which enable us to largely remove the influence from different culture-backgrounds. Second, both have been widely used in measuring the relation between WM and Gf (e.g., Kane et al., 2005; Fukuda et al., 2010; Unsworth et al., 2014). For CCFIT, we adopted a full-scale measure in accordance with previous studies (Fukuda et al., 2010; Unsworth et al., 2014), which is composed of four separate and timed paper-and-pencil sessions (Cattell, 1971). Participants were given about 2∼3.5 min to finish each session. In the first session, participants saw 13 incomplete series of abstract shapes, along with 6 alternatives for each, and selected one that best completed the series. In the second session, participants saw 14 problems composed of abstract shapes, and chose the two out of the five that differed from the other three, e.g., shapes differed in content, orientation, or size. In the third session, participants saw 13 incomplete matrices containing four to nine boxes that had abstract shapes as well as an empty box and six choices. They had to infer the relations among the items in the matrix and select an answer that correctly fulfill each matrix. In the fourth session, participants saw 10 sets of abstract figures consisting of lines and a single dot along with five alternatives. They needed to assess the relation among the dot, figures, and lines, and choose the alternative in which a dot could be placed according to the same relation. The final score of CCFIT was the total number of items solved correctly across all four sessions. For RAPM, which is a measure of abstract reasoning, we chose a split-half measure (Jaeggi et al., 2008; Broadway and Engle, 2010; Shipstead et al., 2012) to shorten total experiment time course to avoid fatigue. A full scale of RAPM was split into odds and even items and each participant had 20 min to complete the split-half scale. Note that in previous studies wherein a split-half measure of RAPM was adopted, researchers usually gave participants 30 min to finish the test. However, a pre-test with a sample of another 10 participants from Zhejiang University resulted in ceiling effect with a 30-min duration, we hence reduced the testing duration to 20 min. Each split-half measure of RAMP consists of 18 items displayed in ascending order of difficulty. Each item consists of a display of 3 × 3 matrices of geometric patterns with the bottom right one missing. Participant had to select one among eight alternatives, which can correctly complete the overall series of patterns. The final score of RAPM was the total number of correct solutions. For both CCFIT and RAPM, participants scored 1 point if they answered correctly on an item and 0 if they were wrong.

#### Procedure

For the BM-WM test, each trial began with two white digits showing in the center of the screen for 500 ms (see Figure 1). Participants were demanded to repeat the two digits (e.g., “six,” “three,” “six,” and “three”) aloud. This manipulation was set to prevent them from verbally processing those movements (cf. Gao et al., 2013; Shen et al., 2014; van Boxtel and Lu, 2015). A red fixation then appeared for 300 ms and, after a blank interval of 150 ms–350 ms, the memory array was presented on the screen for Ns (according to the number of PLDs movements, e.g., 5 s for 5 stimuli; cf. Shen et al., 2014) to avoid underestimating the WM capacity of BM. Following a 1-s blank interval, a red probe appeared in the screen center for 1 s. From then on, participants stopped repeating the digits. The probe then disappeared, followed by a red question mark showing at the screen center, and participants had 3 s to decide whether the probe had appeared in the memory array before by pressing a button to relay the judgment. After the response, or if no response was made within 3 s, a red digit would be presented after a 100 ms delay. Participants had to decide whether the red digit was one of the previously rehearsed digits by pressing the same buttons as above. Participants were told to respond as accurately as possible. There were 30 trials under each memory load, resulting in 150 trials in total. Before the formal experiment, there are 16 trials for participants to practice.

Half of the participants performed the BM-WM measurement before the two Gf measurements and the other half on the opposite, and the two Gf measurements were given to the participants in random order. Before each task, the experimenter would stress to the participant that they needed to try their best, either to remember the stimuli or to answer each item in the two questionnaires. For Gf measurement, participants were instructed to write their answers on an answering sheet and draft papers were provided. The experimenter monitored the time to ensure the task was fulfilled within the required time window. The whole test was around 70 min.

#### Analysis

To estimate BM-WM capacity, we employed Cowan’s formula (Cowan, 2001): *K* = *S* × (*H* - *F*), where *K* is the WM capacity, *S* is the number of to-be-memorized stimuli, *H* is the hit rate that refers to the successful detection of a new stimulus, and *F* is the false alarm rate that refers to an incorrect new-stimulus response. We calculated *K* for each set size of each participant. To have a more accurate estimate, we considered the maximum *K* (*K*-max) among the five load conditions as one’s WM capacity (e.g., Curby and Gauthier, 2007; Gao et al., 2013; Shen et al., 2014; Guo et al., 2019). Only trials with correct responses for digit task were analyzed. Finally, Pearson’s correlations between *K*-max and the scores on the two Gf measurements were calculated.

### Results

Descriptive statistics of each measured variable are shown in Table 1. One-sample Kolmogorov-Smirnov test showed that all the measured variables conformed to normal distributions (*ps* > 0.05; see also the Skewness in Table 1).

**TABLE 1:** Mean value (SE) and results of skewness test of each measured variable in the current study.

	**Statistics**	**WM task (*K*-max)**	**CCFIT Score**	**RAPM Score**
**Pilot study**				
BM	Mean (SE)	3.05 (0.73)	37.37 (5.16)	11.93 (2.67)
	Skewness	0.02	−0.76	−0.15
**Formal study**				
BM	Mean (SE)	3.14 (0.77)	37.40 (4.67)	11.61 (2.73)
	Skewness	−0.01	−0.63	−0.38

**FIGURE 2:**
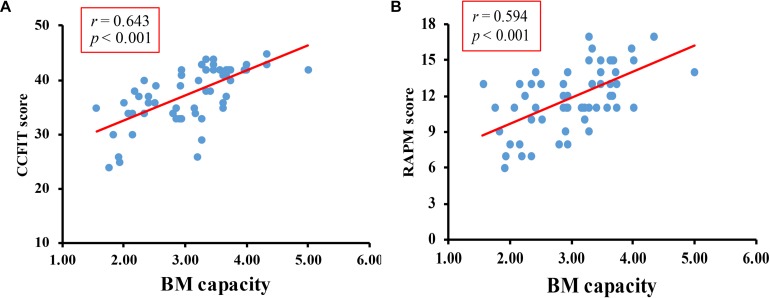
Results of Pilot study. **(A)** The correlation between BM-WM capacity and CCFIT. **(B)** The correlation between BM-WM capacity and RAPM.

The overall accuracy of the digit rehearsal task was 95%. The correlation between CCFIT score and RAMP score was *r* = 0.597, *p* < 0.001. Pearson’s correlation test revealed a significantly positive correlation between *K*-max and CCFIT score (Figure 2A), *r* = 0.643, *p* < 0.001, as well as between *K*-max and the RAPM score (Figure 2B), *r* = 0.594, *p* < 0.001.

### Discussion

Results of our pilot study revealed a significant correlation between BM-WM capacity and Gf, suggesting that the performance of BM-WM can predict one’s cognitive ability. As a small sample size of 60 may not be sufficient to draw a robust conclusion, we used G^∗^power 3.1 to determine our final sample size (Faul et al., 2009). To achieve a medium effect size (*d* = 0.3 for Pearson correlation) and a power of 0.9 at 0.05 significant level, we had to test at least 112 participants. To this end, we tested another 55 participants to ensure our sample size is big enough. All testing procedures were pre-registered with the Open Science Framework^3^.

## Formal Study

### Method

Together with the 60 participants in the pilot study, 115 (60 female; mean ± SD age 20.1 ± 1.7 years) participants took part in the experiment. Eight-five participants were from Zhejiang University and 30 were from Yunnan University and Yunnan Normal University. Participants all had normal or correct-to-normal vision and normal color vision. Participants received payment/course credit for their participation. Two participants were excluded from further analysis because the *K*-max was below 3 standard deviations of the average, which resulted in a final sample size of 113. Before the experiment, participants provided signed informed consent. The study was approved by the Research Ethics Board of Zhejiang University, Yunnan University, and Yunnan Normal University. The stimuli and procedures were all the same as in the pilot study.

### All Results

Descriptive statistics of each measured variable and results of tests for skewness are shown in Table 1.

**FIGURE 3:**
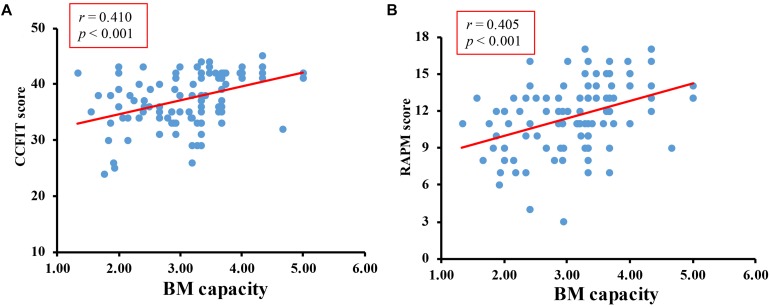
Results of formal study. **(A)** The correlation between BM-WM capacity and CCFIT. **(B)** The correlation between BM-WM capacity and RAPM.

Overall accuracy of the digit rehearsal task was 96%. The correlation between CCFIT score and RAMP score was *r* = 0.579, *p* < 0.001. The correlations between *K*-max and CCFIT, *K*-max and RAPM were *r* = 0.410, *p* < 0.001, and *r* = 0.405, *p* < 0.001, respectively (Figure 3).

## General Discussion

The goal of our study was to examine whether BM-WM capacity can predict canonical cognitive ability. In contrast to the prediction of a null relation between BM-WM and Gf, correlation analysis revealed a significantly positive correlation between BM-WM capacity and the two Gf measurements, suggesting that, although BM processing has an intimate relation with social cognition, the capacity of BM-WM can predict one’s high-level general cognitive ability (Gf).

### Why a Relation Between BM-WM and Gf Exists?

We argue that the reason for an intimate relation between BM-WM and Gf may lie in the neural substrates involved in BM processing. While distinct visual objects (e.g., color and shape) are processed and stored via the primary visual cortex (Harrison and Tong, 2009; Serences et al., 2009), the processing of BM requires the involvement of a much broader brain network. Neuroimaging studies revealed that two visual pathways are engaged in BM processing, with the ventral pathway handling form information while the dorsal pathway addressing motion information (e.g., Giese and Poggio, 2003; Gilaie-Dotan et al., 2011). The two pathways converge in the superior temporal sulcus (STS) to have a coherent representation of BM. Additionally, the MNS is also involved in processing BM (e.g., Rizzolatti and Craighero, 2004; Pineda, 2005; Perry et al., 2010). Along the same lines, recent fMRI studies revealed that both the neural substrates that were dedicated to core social ability (MNS), and those for canonical cognitive processing (SPL, IPL, and bilateral prefrontal cortex), are involved in the retention of BM in WM (Lu et al., 2016; Cai et al., 2018). Therefore, we consider that our previous work showing the relation between BW-WM and empathy reflects the contribution of MNS as well as STS, and the current finding may reflect the contribution of SPL, IPL, and bilateral prefrontal cortex. From this perspective, the current study sheds critical light on future clinical interventions focusing on WM. That is, future clinical interventions might consider training on BM-WM, which might be beneficial to both cognitive and social abilities.

### Implications of the Current Study

The current study is among the first that directly investigates the relationship between BM-WM and Gf, contributing to the BM research field in general and the BM-WM explorations in particular. Although there have been a few studies examining the relationship between BM perception and Gf, the results were mixed (e.g., Barresi and Moore, 1996; Shinkfield et al., 1999; Blake et al., 2003; Rutherford and Troje, 2012). Recently researchers even considered Gf to play a “scaffolding” role in processing BM, i.e., when one’s social ability is impaired, individuals turn to exploit general cognitive processes to handle BM information (Rutherford and Troje, 2012). The current study extended the exploration from perception to WM. In contrast to the implications from BM perception, we presented clear-cut evidence that higher WM capacity of BM predicts a higher IQ score. Therefore, the BM-WM capacity not only correlates to social ability but also has an intimate link with cognitive ability.

The current exploration also shed critical light on the function of social WM. Currently, it has been revealed that human brain has evolved neural substrates dedicated to social WM (e.g., dorsomedial prefrontal cortex, ventromedial prefrontal cortex, and right temporo-parietal junction; Meyer and Lieberman, 2012; Meyer et al., 2012, 2015), which is deactivated during canonical cognitive WM tasks (e.g., memorizing colors, locations, letters). Although previous social WM studies focused on peoples’ trait and emotions (e.g., Meyer and Lieberman, 2012, 2016; Meyer et al., 2012, 2015; Thornton and Conway, 2013; Xin and Lei, 2015), the explorations of social WM should not be constrained to these sets of information. Indeed, the advance of social WM is to emphasize that the canonical WM studies have largely overlooked the temporal storage and manipulation of social information in our life, for instance, people’s identities, mental states, traits, and interpersonal relationships. As we reviewed in the introduction, a bunch of social information (identity, emotion, intention, goal, and gender, etc.) could be extracted even from PLD format BM, and one’s recognition ability of BM is taken as a hallmark of social cognition (Pavlova, 2012), which, to the best of our knowledge, is the only stimuli category receiving this evaluation in terms of measuring social ability. To this end, we consider that BM is a well-matched stimulus in measuring social WM, and we used it to measure the development of social WM in 3∼6 preschoolers (He et al., 2019). Taking all the related explorations of social WM together (i.e., using people’s trait, emotion, and BM as the stimuli of interest), we noticed that the extant studies on social WM mainly focused on the storage capacity and manner (Shen et al., 2014; Gao et al., 2016; He et al., 2019; Liu et al., 2019), and neural substrates of social WM (Lieberman, 2007; Meyer and Lieberman, 2012, 2016; Meyer et al., 2012, 2015; Thornton and Conway, 2013; Xin and Lei, 2015; Lu et al., 2016). A few studies had attempted to explore the functions of social WM (Meyer et al., 2012, 2015; Xin and Lei, 2015; Gao et al., 2016). However, to date, all of related studies focused on the relation of social WM to social abilities. The current study closed a key gap when uncovering the function of social WM, and implied that, although social WM had special neural substrates from canonical cognitive WM (e.g., object WM), there were no double dissociations in terms of different roles of WM buffers (i.e., canonical cognitive WM links to cognitive ability and social WM links to social ability). Instead, akin to canonical cognitive WM, the capacity of social WM (at least for certain representatives) has a close relationship with Gf.

### Limitations & Future Studies

The current study aimed at exploring the function of BM-WM by exploring the relationship between BM-WM capacity and Gf using a correlation analysis. To have a comprehensive understanding of the function of BM-WM, we argue that at least two aspects have to be addressed in future studies. First, additional study is needed to further examine the relation between BM-WM capacity and Gf, for instance, by using different testing procedures (e.g., a recall task of WM, Zhang and Luck, 2008) and sample selections (e.g., using students in primary or middle school). Moreover, the current experiment essentially used a dual-task setting (i.e., an articulatory suppression task and a WM task), which has been widely used in both BM-WM and object-WM fields to measure the WM capacity. Future study may consider to partial out the effect of articulatory suppression, such that we can have a pure estimation of the relation between BM-WM capacity and Gf. Second, Gao et al. (2016) and the current study explored the function of BM-WM from the perspective of social ability and cognitive ability, respectively. Moreover, both studies used a correlational analysis. To have a full picture of the function of BM-WM and to figure out the underlying relation between social and cognitive abilities, it would be more informative to put all the related factors (e.g., empathy, Gf, BM-WM capacity, object-WM capacity, and attention) in one study, and perform more comprehensive analysis such as latent variable analysis (e.g., Unsworth et al., 2014).

Additionally, based on the previous studies (Smyth et al., 1988; Smyth and Pendleton, 1989, 1990; Wood, 2007, 2008, 2011; Cortese and Rossi-Arnaud, 2010; Shen et al., 2014; Liu et al., 2019), the current study claimed that BM has an independent buffer in WM in terms of Baddeley and Hitch (1974). It is worth noting that processing (including perception and WM) human body-related images (e.g., hand gesture) also activates MNS (Mecklinger et al., 2002; Moreau, 2013; Galvez-Pol et al., 2018a, b; Arslanova et al., 2019), hence, it is also possible that the currently tapped BM-WM is actually an independent buffer dedicated to maintaining body-related stimuli regardless of motion (BM is just one example). However, we argue that it is premature to claim an independent WM buffer for body-related stimuli, considering that all related studies on the storage buffer of BM in WM focused on dynamic BM (Smyth et al., 1988; Smyth and Pendleton, 1989, 1990; Wood, 2007, 2009, 2011; Cortese and Rossi-Arnaud, 2010; Shen et al., 2014; Liu et al., 2019). Indeed, there are at least two reasons against the use of this independent WM buffer for body-related stimuli in general. First, the processing of BM is more complex than a single body-related image in terms of both cognitive and neural processing. For cognitive processing, the formation of a coherent BM representation requires our cognitive system to integrate different pieces of information (i.e., individual frames or images) across space and time (Orgs and Haggard, 2011). Lange and Lappe (2006) suggested that BM perception is achieved by dynamically integrating the activity of template cells of static form information the human body (i.e., body image), and this process requires the help of attention (Thornton et al., 2002; see Thompson and Parasuraman, 2012 for a review). For neural processing, unlike perceiving hand or face images which usually activates more posterior cortices, such as somatosensory cortices, extrastriate body area, and fusiform (Kanwisher et al., 1997; McCarthy et al., 1997; Gauthier et al., 2000; Galvez-Pol et al., 2018a, b; Arslanova et al., 2019), BM perception and WM maintenance activate more anterior regions, such as superior temporal sulcus, inferior frontal gyrus and ventral premotor cortex (e.g., Perry et al., 2010; Lu et al., 2016; Cai et al., 2018). Second, according to the core knowledge architecture of visual WM of Wood (2011), BM and body-related image should be stored in different buffers. This architecture claims that there are distinct buffers in visual WM for retaining spatiotemporal information (for object tracking, e.g., BM), object property/kind information (for object recognition, e.g., the form of a BM stimulus), and view-dependent snapshot information (for place recognition; e.g., four distinct colors in a 2D space). The dynamic BM belongs to spatiotemporal information, while body-related image belongs to view-dependent snapshot information. To this end, we consider that it is important to examine whether there is an independent WM buffer for body-related stimuli, by requiring participants to memorize dynamic BM and static body-related stimuli in one task.

## Data Availability Statement

All datasets generated for this study are included in the manuscript/supplementary files.

## Ethics Statement

The studies involving human participants were reviewed and approved by the Research Ethics Board of Zhejiang University, Yunnan University, and Yunnan Normal University. The patients/participants provided their written informed consent to participate in this study.

## Author Contributions

ZG put up the question and designed the experiment. TY and PL collected the data. QZ helped with preparing IQ measurements. TY wrote the first draft of the manuscript. ZG made critical changes on it. QG, XL, and MS provided the meaningful suggestions on the final version of the manuscript.

## Conflict of Interest

The authors declare that the research was conducted in the absence of any commercial or financial relationships that could be construed as a potential conflict of interest.

## Footnotes

1 It is of note that there are two distinct views as to the unit of VWM (i.e., slots vs. resources; Bays and Husain, 2008; Zhang and Luck, 2008; see Suchow et al., 2014 for a review). So far, the available studies implicitly assumed that slots are basic units in VWM for storing BMs. Here we reviewed and summarized these results. The unit of storing BMs in VWM is beyond the scope of current work.

2 The term WM used here is identical to the system that is often called short-term memory. Considering most of the extant studies using change detection task to explore the mechanisms of short-term memory adopted the term WM, we followed the previous studies and used the term WM.

3 https://osf.io/cqkx6/
